# The impact of rising global temperatures on neurological diseases

**DOI:** 10.1055/s-0046-1829128

**Published:** 2026-10-27

**Authors:** Gabriela Monteiro Pereira, Gustavo Wagner Baratti Rocha, Li Min Li

**Affiliations:** 1 Universidade Estadual de Campinas, Faculdade de Ciências Médicas, Departamento de NeurologiaCampinas SPBrazil

**Keywords:** Climate Change, Global Warming, Nervous System Diseases, Infrared Rays

## Abstract

**Background:**

Climate change, driven predominantly by anthropogenic actions, represents a defining challenge of the 21st century. While its general health implications are well documented, the specific impact of global warming on neurological diseases has remained underemphasized for decades.

**Objective:**

To evaluate the association between rising global temperatures and the exacerbation or incidence of various neurological conditions through a systematic review.

**Methods:**

A systematic search was conducted in the PubMed/MEDLINE and SciELO databases for studies published between 2011 and 2026. Following strict eligibility criteria, 13 peer-reviewed articles were selected for qualitative synthesis.

**Results:**

The evidence reveals that rising temperatures are increasingly linked to the worsening of conditions such as epilepsy, stroke, multiple sclerosis (MS), and neurodegenerative disorders like Alzheimer's and Parkinson's diseases. Specifically, heat exposure is associated with higher hospitalization rates and acute symptom exacerbation, such as Uhthoff's phenomenon in MS and autonomic thermoregulatory failure in Parkinson's. Although some geographic and methodological discrepancies exist, particularly regarding stroke and seizure frequency, the overall trend indicates increased neurological vulnerability to thermal stress.

**Conclusion:**

While research is expanding, neurological diseases remain an understudied frontier of the climate crisis. Targeted scientific investigation and robust public health strategies are imperative to mitigate the emerging neurological risks of a warming planet.

## INTRODUCTION

Climate change is considered the greatest scientific and medical challenge of the 21st century. 1 2 Alongside other environmental changes, such as ozone layer depletion, pollution, and urbanization, climate change is a threat to the planet and human health. Three components are involved in the definition of climate change: global warming with sea-level rise and acidification, alteration of precipitation patterns, and increased occurrence of extreme weather events. 1

Global warming is characterized by the increase in Earth's temperature and is caused by the intensification of the greenhouse effect. 2 3 The greenhouse effect, in turn, is characterized by the partial retention of thermal infrared radiation, which is driven by the so-called greenhouse gases: carbon dioxide, methane, and nitrous oxides. This current intensification has occurred mainly due to anthropogenic actions. 3

Since the 18th century, emissions of carbon dioxide and other gases have increased. Since the 1950s, scientific evidence has pointed to the possibility of global climate change due to the altered atmospheric concentration of the aforementioned gases, 3 given that the Earth's average temperature has increased by 1.48 °C since the Industrial Revolution. 4 The Intergovernmental Panel on Climate Change (IPCC) was created to monitor and quantify these climatic alterations. In the first report, in 2001, carbon concentration reached its highest level in the past 400,000 years. The third report, however, showed that the exacerbated emission of greenhouse gases is closely related to anthropogenic actions, information confirmed by the fourth report (2007). 3 5 It is worth noting that 2023 has been the hottest year on record thus far. 4

Currently, human health problems related to climate change and heat waves are highly prevalent topics in the current scientific community, given that many people are already affected by an increase in morbidity and mortality due to global warming. An analysis of lethal events documented in 36 countries from 1980 to 2024 by Mora et al. 6 revealed a threshold beyond which the mean temperature and air humidity become lethal. However, the impact of heat on neurological patients has been an understudied topic until now, and it was only in 2011 that the scientific committee of the World Federation of Neurology added the neurological consequences of global warming to its discussion agendas. 7

Since 2021, several researchers have placed greater focus on the impact of the climate crisis on neurological diseases, such as stroke, Parkinson's disease (PD), Alzheimer's disease (AD), amyotrophic lateral sclerosis (ALS), epilepsy, and multiple sclerosis (MS). Furthermore, alterations in the circadian rhythm and the human glymphatic system were addressed in the studies. 8 The rise in temperature impacts multiple mechanisms. Heat directly affects molecular functions, especially those of ion channels. Heat can both decrease the pharmacological effect of medications and aggravate the pathophysiology of neurological diseases. During the 2023 heatwave in Europe, it was estimated that 14,539 excess deaths occurred in France, of which 3,377 (23.2%) were due to stroke, hyperthermia, and diseases of the nervous system or mental disorders. 4

Finally, neurological disorders are a leading cause of disability-adjusted life years (DALYs) and death worldwide, 4 and to establish an association between neurological diseases and the rise in global temperature is of utmost importance.

## METHODS

### Eligibility criteria

#### *Inclusion*

A search for articles was conducted looking for the impacts of climate change on human neurological diseases. Eligible papers must have been written in Portuguese or English languages and published between January 1, 2011, and January 1, 2026.

#### *Exclusion*

Records were excluded if they consisted of duplicates, studies with inadequate designs, abstracts, or unpublished manuscripts. Furthermore, articles that did not have the discussed indicators as their primary focus or did not focus specifically on human neurological disorders were removed. Studies without a clear conclusion or those that only raised theoretical questions without providing data were also excluded. To minimize methodological variability, the review omitted research referring to historical climatic events over millennia rather than current climate change, as well as articles that did not address specific neurological diseases but focused on non-specific impairments, such as general motor performance.

### Search strategy and data collection

The main objective to be reached was to establish whether there is an association between neurological diseases and the rise in global temperature. The literature search was conducted in the PubMed/MEDLINE and SciELO databases. The last search was performed on March 19, 2026. Controlled vocabulary and free-text terms related to ( *global warming* AND *central nervous system diseases* ) OR (“ *climate emergency and neurology* ) OR ( *effects of global warming on the brain* ) were combined using Boolean operators. All terms were searched in both Portuguese and English.

Two reviewers (GMP and GWBR) independently screened the articles, and any discrepancies were resolved by consensus. A total of 147 records were identified in the databases, all of which were retrieved from PubMed, with no additional results found in SciELO. Following the application of inclusion and exclusion criteria, 16 articles were selected for full-text assessment. Among these, three were excluded for failing to correlate global warming with impacts on neurological health. Ultimately, 13 articles met all eligibility criteria for this review ( Table 1 ). The included studies were conducted in diverse geographic locations, including the United States, Western Europe, China, Australia, Brazil, Canada, and South Korea. Finally, the analysis focused on the most prevalent neurological conditions: stroke, Parkinson's disease (PD), Alzheimer's disease (AD), amyotrophic lateral sclerosis (ALS), epilepsy, and multiple sclerosis (MS).

**Table 1:** Assessment of individual studies

Article	Conclusion
Ruszkiewicz et al. (2019) 1	Climate change may disrupt critical public health factors, such as water quality and environmental pollution, thereby impacting risk factors for brain diseases.
Sisodiya et al. (2019) 2	Climate change affects human disease risks and healthcare delivery, necessitating further investigation.
Amiri et al. (2021) 12	The 93 included studies showed methodological heterogeneity but indicate that climate change affects all major neurological disorders.
Gulcebi et al. (2021) 13	Climate change has a multifactorial association with epilepsy, varying according to specific seizure characteristics.
Bongioanni et al. (2021) 16	Suggests the hypothesis that heat exacerbates molecular mechanisms in neurodegenerative diseases.
Louis et al. (2023) 11	A review of 364 articles highlighted an association between climate change and the exacerbation of neurological symptoms.
Ranta et al. (2023) 15	Environmental factors, such as air pollution, atmospheric brown clouds, and desert dust storms, influence stroke risk.
Hameed et al. (2024) 8	Short-term exposure to air pollution appears to be associated with an increased risk of stroke.
Ranta et al. (2024) 14	There is consistent evidence linking air pollution and elevated temperatures to stroke incidence.
Sisodiya et al. (2024) 4	Evidence suggests that climate change affects the incidence, prevalence, and severity of nervous system conditions.
Reis et al. (2024) 6	Heat waves appear to significantly impact patients with preexisting neurological disorders.
Yang et al. (2025) 17	Global warming is particularly aggravating for the incidence and severity of heatstroke.
Khalsa et al. (2025) 18	Air pollution and global warming contribute to an elevated risk of Alzheimer's disease.

### Quality assessment and risk of bias

The methodological quality of the studies included was assessed with the Joanna Briggs Institute Critical Appraisal tools for systematic reviews 9 for those studies employing a systematic design. For the remaining articles which lack the methodological prerequisites for systematic appraisal, we assigned a *level 5* classification according to the Oxford Centre for Evidence-Based Medicine (OCEBM) 10 levels of evidence, acknowledging their inherent risk of selection and reporting bias. The overall body of evidence remains heterogeneous, particularly due to the challenges in environmental epidemiology, such as the difficulty in controlling for all confounding environmental variables (what was considered a climate change), and variable eligibility criteria, which contributes to an overall moderate risk of bias across the literature ( Table 2 ).

**Table 2:** Risk of bias based on JBI and OCEBM

Article	Q1	Q2	Q3	Q4	Q5	Q6	Q7	Q8	Q9	Q10	Q11
Ruszkiewicz et al. (2019) 1	Level 5 Evidence (Expert Opinion)										
Sisodiya et al. (2019) 2	Level 5 Evidence (Narrative Review)										
Amiri et al. (2021) 12	Yes	Yes	Yes	Yes	Yes	Yes	Yes	Yes	No	Yes	Yes
Gulcebi et al. (2021) 13	Level 5 Evidence (Narrative Review)										
Bongioanni et al. (2021) 16	Level 5 Evidence (Narrative Review)										
Louis et al. (2023) 11	Yes	Yes	Yes	Yes	Yes	Yes	Yes	Yes	No	Yes	Yes
Ranta et al. (2023) 15	Level 5 Evidence (Narrative Review)										
Hameed et al. (2024) 8	Level 5 Evidence (Narrative Review)										
Ranta et al. (2024) 14	Level 5 Evidence (Narrative Review)										
Sisodiya et al. (2024) 4	Level 5 Evidence (Expert Opinion)										
Reis et al. (2024) 7	Level 5 Evidence (Narrative Review)										
Yang et al. (2025) 17	Level 5 Evidence (Narrative Review)										
Khalsa et al. (2025) 18	Level 5 Evidence (Narrative Review)										

Abbreviations: JBI, Joanna Briggs Institute; OCEBM, Oxford Centre for Evidence-Based Medicine.

Notes: Q1–Q11 are from the JBI Critical Appraisal Checklist for Systematic Reviews and Research Syntheses applied to systematic review design studies. Narrative reviews and expert opinion articles were classified according to the OCEBM Levels of Evidence.

However, a distinction was made for the large-scale systemic analyses. The studies by Louis et al. (2023) 11 and Amiri et al. (2021), 12 which involved extensive case series, demonstrated a low risk of bias. These works utilized rigorous screening protocols and standardized data extraction methods, with high-quality evidence reported in the current review.

### Qualitative synthesis

Data extraction was focused on three primary thematic axes: a) the direct impact of ambient temperature on disease incidence and acute exacerbation; b) the synergistic effects of global warming and environmental pollutants on neurodegeneration; and c) the physiological mechanisms of heat vulnerability in patients with preexisting neurological conditions.

Large-scope analyses were the fundamental basis of this review. Overall, the conclusions of the assessed articles were taken into consideration. The synthesized data was categorized by disease phenotype (epilepsy, stroke, multiple sclerosis, and Parkinson's and Alzheimer's diseases) to clarify how heat exposure disrupts specific neurophysiological pathways. This approach allowed for a comprehensive overview of the current evidence while highlighting the conflicting results found in certain pathologies, such as ischemic stroke and seizure frequency.

### Ethical considerations

As the current study is a systematic review of previously published literature and did not involve direct interaction with human participants or the use of identifiable private information, it was exempt from Institutional Review Board (IRB) approval. The present systematic review followed the Preferred Reporting Items for Systematic Reviews and Meta-Analyses (PRISMA) guidelines to ensure transparency and methodological rigor ( Figure 1 ). Although the study was not prospectively registered in the International Prospective Register of Systematic Reviews (PROSPERO) database, as the initial project phase was exploratory in nature, the finalized review protocol, including the research question, predefined search strategy, and strict inclusion/exclusion criteria, was established a priori to prevent reporting bias and ensure the reproducibility of our findings. All the studies included were previously approved by their respective ethics committees, and the authors of the current review declare no violations of ethical conduct during data extraction and synthesis.

**Figure 1:**
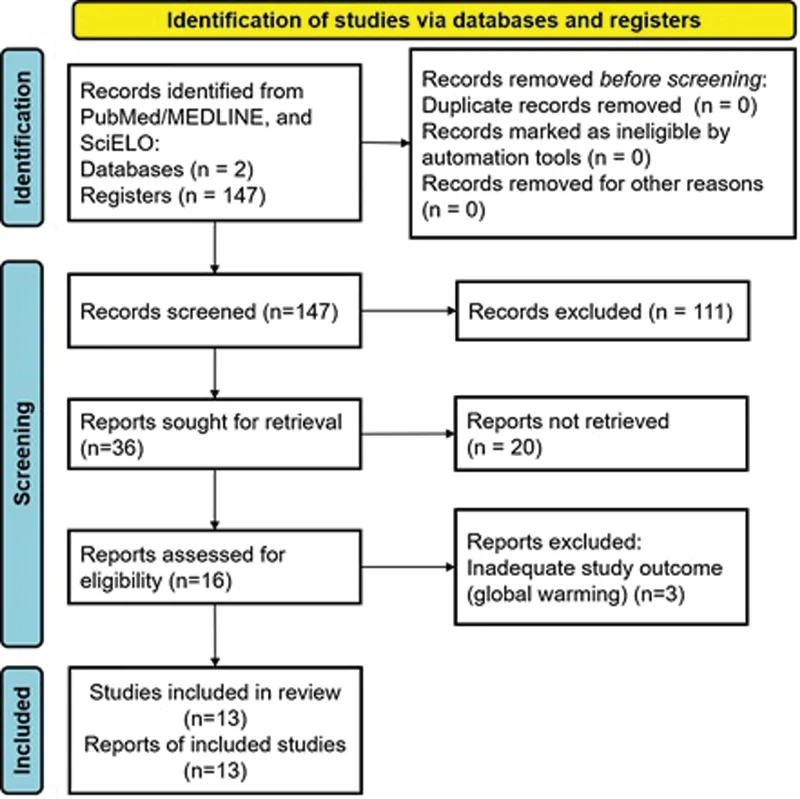
Preferred Reporting Items for Systematic Reviews and Meta-Analyses (PRISMA) flowchart.

## RESULTS

### Results of assessed studies and their general impact

The systematic analysis reveals a consistent correlation between climate change, primarily global warming and air pollution, and the exacerbation of neurological conditions. Sisodiya et al. 2 emphasized that these changes pose a dual threat, affecting both patient health risks and the operational capacity of healthcare providers, thus requiring more focused research. Sisodiya et al., 4 5 years later, published that there is evidence that environmental shifts influence not only the severity of nervous system diseases but also their incidence and prevalence. 4 Gulcebi et al. 13 highlighted a multifactorial association, noting that the impact varies according to the specific characteristics of the seizure disorder.

The impact on cerebrovascular health was a prominent theme. Ranta et al. 14 15 identified that environmental factors, including fine particulate matters and rising temperatures, are possibly associated with an increased incidence of stroke. This is complemented by the findings of Hameed, 8 which specifically linked short-term air pollution to acute stroke risk.

The public health implications were addressed by Ruszkiewicz et al., 1 who showed how climate change disrupts essential environmental factors, such as water and air quality, intensifying risk factors for brain disorders. Reis et al. 7 demonstrated that heat waves specifically act as critical stressors for patients with preexisting neurological impairments.

Large series, such as the one by Amiri et al., 12 despite noting significant methodological heterogeneity among its 93 studies, concluded that climate change adversely affects all major neurological disorders. In the same direction, a review of 364 articles by Louis et al. 11 underscores that climatic shifts are directly linked to the worsening of neurological symptoms across various populations.

### Multiple sclerosis (MS)

Studies conducted between 2003 and 2017 in the United States found that abnormal heat (more than 1.5 °C above the long-term monthly average temperature) was associated with an increase in emergency department visits and inpatient admissions. Thus, individuals with MS may represent a susceptible subpopulation. 4 7

Furthermore, individuals with MS naturally exhibit heat sensitivity (Uhthoff's phenomenon). Increases in body temperature induced by exercise or passive heating trigger a temporary worsening of clinical symptoms (cognitive, motor, visual impairment, and fatigue) in 60 to 80% of patients with MS. 4 7 This occurs due to impaired action potential propagation in demyelinated axons, along with an abnormal sweat distribution and an attenuated sweating response, whereas the vasomotor response is maintained. 7 Additionally, thermal dysregulation due to cerebrospinal fluid (CSF) diffusivity alteration contributes to the aggravation of the symptoms. 7

In Europe, in 2003, heat waves did not appear to increase the number of hospital admissions or relapses in MS patients compared with the 3 previous years. However, mortality rates among patients with MS in the US are temperature-dependent, and heat may be associated with an increase in this rate. 7 The temperature-related worsening of symptoms can cause short-term “relapses” without neuroradiological changes. 4 12

### Parkinson's disease (PD)

Patients with PD exhibit thermoregulatory alterations due to autonomic failure (abnormal sweating to reduce body temperature). Thus, they may be susceptible to temperature increases. The heat waves from 2008 to 2009 in Spain were linked to an 11.5% increase in daily emergency hospital admissions and a 12% increase in mortality for every degree of ambient temperature above 34 °C among patients with PD. Epidemiological data from 185 countries revealed a positive association between intense ambient heat and the prevalence of PD, with the impacts of climate crisis on the rates of this disease being greater in countries with hotter climates. 7 However, some studies on PD and heat have shown conflicting results. 12

### Alzheimer's disease (AD)

Alzheimer's disease is the most prevalent neurodegenerative disease and is linked to functional disturbances; the circadian rhythm, sleep patterns, cognition, and thermoregulation. It is believed that the impairment of thermoregulatory function in older adults is exacerbated in AD, leading to a thermoregulatory and metabolic deficit. However, the relationship between body temperature and neurocognitive health remains unknown. 7

A retrospective mortality study among elders over 74 years during the 2003 heatwave period in Italy showed that cerebrovascular diseases and dementia were the main causes of increased mortality. Another retrospective study revealed that the risk of dementia-related hospitalizations in England increased by 4.5% for each degree rise on the ambient temperature above 17 °C. Furthermore, an Australian retrospective cohort study of hospitalized patients with AD from 2005 to 2013 confirmed that heat waves increase the risk of hospitalizations and post-discharge deaths within a 2-month period. 7

Nine out of 11 studies on AD and other types of dementia, conducted across 8 countries, showed high temperatures were associated with the worsening of symptoms in these patients and an increase in hospitalization and mortality. 12 Additionally, two studies showed a relationship between an increased number of hospitalizations of patients with dementia during the summer. 15

### Stroke

Stroke is one of the leading causes of death and disability worldwide. 4 8 15 Furthermore, a 70% increase in incidence has been observed over the past 30 years. 8 Climate change may influence the incidence or outcome of stroke, but the data are inconclusive: studies from the US and South Korea have shown an increase in the incidence of stroke with rising temperatures and relative humidity extremes, whereas studies in Canada and Spain found an increase in stroke admissions at lower temperatures. 4

A Chinese study from the 2014-to-2019 period revealed that both heat waves and cold spells affected blood pressure and the coagulation system. Within 24 hours of hospital admission, patients with ischemic stroke ( *n* = 806) who experienced heat waves showed increased blood pressure and altered coagulation parameters (reduced thrombin time and activated partial thromboplastin time). Moreover, dehydration also increases the risk of microvascular thrombosis. Although these alterations may contribute to the increased risk of stroke during heat waves, further studies are needed to better establish this relationship. 7

In summary, the evidence regarding the relationship between rising temperatures and stroke occurrence is not definitive. Most studies show an association with increased incidence and mortality, 12 14 15 but there are also studies contradicting this assertion, demonstrating an association between increased ischemic stroke (IS) at lower temperatures. 8 11 15 At lower temperatures, the higher occurrence of IS may be associated with vasoconstriction and increased blood viscosity. 13 15 This discrepancy could be indicating bimodal (U curve) presentation with association of stroke to the extreme temperature variation.

### Epilepsy

Approximately 1% of the world population (70 million people) have epilepsy. Most patients with epilepsy present characteristics that are likely to be exacerbated by climate change, such as sensitivity to sleep deprivation as a seizure precipitant. This deprivation can be worsened by heat waves. 1

There is no clear relationship between exposure to high temperatures and seizure activity in patients with epilepsy, although they appear to be associated. 2 During the Brazilian summers between 2000 and 2015, an increased risk of hospitalization for epileptic seizures related to ambient heat crises was observed: every 1 °C increase above 26 °C raised the risk of hospitalization by 4.3%. 7 There are also studies demonstrating a relationship between the occurrence of seizures and exogenous hyperthermia. 1 11 13 However, a time-stratified case-crossover study from 2010 to 2019 using a US database found no relationship between ambient heat and the risk of an emergency department visit for epilepsy. 7 Finally, some research has even indicated a 46% decrease in the risk of admission for epileptic seizures one day after exposure to temperatures above 20 °C. 12

Thus, the data also indicate a bimodal relationship: some studies report a higher incidence of seizures in epileptic patients at higher temperatures, while others indicate this occurs on colder days. 4

## DISCUSSION

### Assessment of data

The synthesized data revealed significant methodological heterogeneity across the studies included. This divergence is primarily driven by the lack of a standardized definition for *climate change* or *extreme heat events* within the literature. Furthermore, a critical assessment highlights a profound scarcity of systematic reviews specifically dedicated to the intersection of neurology and climatology, explicitly identified in the articles analyzed. There is also a notable deficiency in human cohort studies regarding this theme, which limits the ecological validity of current reviews and potentially hinders an accurate representation of real-world clinical impacts. 16

### Relationship between global warming and neurological pathologies

Overall, the articles analyzed indicated an adverse relationship between global warming and neurological pathologies. A review conducted by Cramer et al. on heat-induced thermoregulatory alterations demonstrated a deleterious effect of global warming on patients with neurological disorders. 4 7 Another study also identified the heat vulnerability of patients with neurological conditions (such as epilepsy, stroke, MS, PD, AD and ALS), who exhibit a lower capacity for physiological adaptation. 7 16

In summary, heat exposure induces complex metabolic changes in the brain: the formation of pathogenic cascades, including heat-induced oxidative stress, endoplasmic reticulum stress, mitochondrial dysfunction, apoptosis, excitotoxicity, neuroinflammation, and impaired cerebral microcirculation, all of which are implicated in neurodegeneration and other brain diseases. 1 16

Overall, the findings of the current systematic review underscore the emergence of new questions regarding pathological conditions resulting from the climatic events of the current century. Climate changes can exacerbate various neurological conditions and impair patients' quality of life. Therefore, new planning strategies are necessary to fight these adverse consequences.

However, despite the growing number of publications on climate and health, there is a general paucity of data on neurological diseases. Several factors contribute to this: interpretations limited by complexities (such as disease heterogeneity), the effect of treatments on climate associations, different disease definitions, and varied methodologies. 4 Furthermore, there is the interaction of global warming with other components of the climate crisis (e.g., air pollution, particulate matters, altered air humidity, healthcare logistics, among others).

These results are useful for formulating public health policies, helping to mobilize resources for sectors that are gaining greater importance. They also assist healthcare professionals in becoming better equipped to handle the newly emerging pathological conditions.

## Study limitations

The assessed data revealed methodological heterogeneity across the studies. Furthermore, a substantial portion of the initial research identified was not focused on human subjects, which complicated the assessment of clinical data directly applicable to patient care. These factors0, combined with the varying metrics used to measure environmental stressors, limit the ability to perform a quantitative meta-analysis and underscore the need for more standardized research protocols in environmental neurology.

In conclusion, although research on heat adaptation in healthy adults has advanced, there remains a significant knowledge gap regarding the specific impacts of climate change on patients with neurological diseases, particularly due to the scarcity of longitudinal cohort studies. While all articles assessed reached a consensus that climate change contributes to an elevated risk of neurological disorders, few provided robust, quantifiable data to establish definitive clinical thresholds. Global warming is expected to disproportionately increase the vulnerability of patients residing in large urban centers. Ultimately, medical preparedness for treating these vulnerable populations is of paramount importance. Expanding the body of evidence through standardized research is essential to ensure that the medical community is adequately equipped to manage this emerging public health crisis in the 21st century.

### Use of Artificial Intelligence

The authors declare that no artificial intelligence tools were used in the preparation of this manuscript.

## References

[1] Ruszkiewicz J A Tinkov A A Skalny A V Brain diseases in changing climate Environ Res 2019 177 108637 10.1016/j.envres.2019.108637 PMC6717544 31416010

[2] Sisodiya S M Fowler H J Lake I Climate change and epilepsy: Time to take action Epilepsia Open 2019 4 04 524 536 10.1002/epi4.12359 PMC6885657 31819908

[3] Lacerda F Nobre P Aquecimento global: conceituação e repercussões sobre o Brasil Rev Bras Geogr Fis 2010 3 01 14–17 10.26848/rbgf.v3i1.232653

[4] Sisodiya S M Gulcebi M I Fortunato F Climate change and disorders of the nervous system Lancet Neurol 2024 23 06 636 648 10.1016/S1474-4422(24)00087-5 38760101

[5] Marengo J Alves L Valverde M Rocha R Laborbe R Eventos extremos em cenários regionalizados de clima no Brasil e América do Sul para o Século XXI: Projeções de clima futuro usando três modelos regionais American Journal of Climate Change 2015 4 03 187 2022007 10.4236/ajcc.2015.43015

[6] Mora C Dousset B Caldwell I Cone C J Rollins E Makela N Global risk of deadly heat Nat Clim Chang 2017 7 07 501 6

[7] Reis J Buguet A Radomski M Stella A B Vásquez T C Spencer P S Neurological patients confronting climate change: A potential role for the glymphatic system and sleep J Neurol Sci 2024 458 122900 10.1016/j.jns.2024.122900 38310733

[8] Hameed S Karim N Wasay M Venketasubramanian N Emerging Stroke Risk Factors: A Focus on Infectious and Environmental Determinants J Cardiovasc Dev Dis 2024 11 01 19 10.3390/jcdd11010019 PMC10816862 38248889

[9] Munn Z Barker T H Moola S Methodological quality of case series studies: an introduction to the JBI critical appraisal tool JBI Evid Synth 2020 18 10 2127 2133 10.11124/JBISRIR-D-19-00099 33038125

[10] OCEBM Levels of Evidence Working Group The Oxford 2011 Levels of Evidence [Internet] Oxford Oxford Centre for Evidence-Based Medicine 2011[cited 2026 Jun 7]. Available fromhttps://www.cebm.ox.ac.uk/files/levels-of-evidence/cebm-levels-of-evidence-2-1.pdf

[11] Louis S Carlson A K Suresh A Impacts of Climate Change and Air Pollution on Neurologic Health, Disease, and Practice: A Scoping Review Neurology 2023 100 10 474 483 10.1212/WNL.0000000000201630 PMC9990849 36384657

[12] Amiri M Peinkhofer C Othman M H De Vecchi T Nersesjan V Kondziella D Global warming and neurological practice: systematic review PeerJ 2021 9 e11941 10.7717/peerj.11941 PMC8349167 34430087

[13] Gulcebi M I Bartolini E Lee O Epilepsy Climate Change Consortium Climate change and epilepsy: Insights from clinical and basic science studies Epilepsy Behav 2021 116 107791 10.1016/j.yebeh.2021.107791PubMed PMC9386889 33578223

[14] Ranta A Kang J Saad A Climate Change and Stroke: A Topical Narrative Review Stroke 2024 55 04 1118 1128 10.1161/STROKEAHA.123.043826 38436104

[15] Ranta A Ozturk S Wasay M Giroud M Béjot Y Reis J Environmental factors and stroke: Risk and prevention J Neurol Sci 2023 454 120860 10.1016/j.jns.2023.120860 37944211

[16] Yang W Gao Z Wang C Heat stroke-induced central nervous system injury: Mechanisms and therapeutic perspectives J Therm Biol 2025 132 104263 10.1016/j.jtherbio.2025.104263 40925107

[17] Bongioanni P Del Carratore R Corbianco S Climate change and neurodegenerative diseases Environ Res 2021 201 111511 10.1016/j.envres.2021.111511 34126048

[18] Khalsa D S Lavretsky H Reddy M Y Fenn A Walling C Air pollution and Alzheimer's disease prevention: The science and a prevention plan J Alzheimers Dis 2025 108 03 953 968 10.1177/13872877251386482 PMC12647382 41134977

